# Deoderized Tincture of Opium

**Published:** 1866-07

**Authors:** 


### Deoderized Tincture of Opium.

We have received from W. H. Peabody, a specimen of deoderized tincture of opium, prepared according to the formula, from the last edition of the United States Dispensatory, in speaking of which it says: "This is an excellent preparation of opium, calculated to supersede various extra-officinal *elixirs* or *solutions*, which have had more or less vogue, based upon the real advantages they afforded in offering liquid preparations of opium exempt from certain noxious ingredients in the crude drug, and in the officinal tinctures, which rendered them so offensive to some constitutions, and in some conditions of disease, as almost to forbid their use. A liquid, watery extract is first made, in which are left behind all the ingredients of opium, soluble in alcohol, and not in water; and this being well shaken with ether, is further deprived of all the principles soluble in this fluid, including narcotina, and the noxious odorous matter, which is probably one of the most offensive and least useful constituents of opium. The ether is then entirely separated, and the residue having been dissolved in water, the solution is filtered and mixed with enough alcohol to preserve it. It may be used in all cases in which laudanum is indicated, but in which it cannot be used in consequence of idiosyncrasy of the patient, or peculiarity in the disease. The dose is the same as that of the officinal tincture of opium."

